# COVID‐19 and maternal and child food and nutrition insecurity: a complex syndemic

**DOI:** 10.1111/mcn.13036

**Published:** 2020-06-16

**Authors:** Rafael Pérez‐Escamilla, Kenda Cunningham, Victoria Hall Moran

**Affiliations:** ^1^ Department of Social and Behavioral Sciences Yale School of Public Health New Haven Connecticut USA; ^2^ Helen Keller International Kathmandu Nepal; ^3^ School of Community Health and Midwifery University of Central Lancashire Preston UK

**Keywords:** COVID‐19, food insecurity, food systems, health care systems, pandemic

## Abstract

Globally, the COVID‐19 pandemic has already led to major increases in unemployment and is expected to lead to unprecedented increases in poverty and food and nutrition insecurity, as well as poor health outcomes. Families where young children, youth, pregnant and lactating women live need to be protected against the ongoing protracted pandemic and the aftershocks that are very likely to follow for years to come. The future wellbeing of the vast majority of the world now depends on reconfiguring the current ineffective food, nutrition, health, and social protection systems to ensure food and nutrition security for all. Because food, nutrition, health, and socio‐economic outcomes are intimately inter‐linked, it is essential that we find out how to effectively address the need to reconfigure and to provide better intersecoral coordination among global and local food, health care, and social protection systems taking equity and sutainability principles into account. Implementation science research informed by complex adaptive sytems frameworks will be needed to fill in the major knowledge gaps. Not doing so will not only put the development of individuals at further risk, but also negatively impact on the development potential of entire nations and ultimately our planet.

Globally, the COVID‐19 pandemic has already led to major increases in unemployment (International Labour Organization, 2020a) and is expected to lead to unprecedentedincreases in poverty (International Labour Organization, 2020b; International Monetary Fund, 2020), as well as poor physical and mental health. COVID‐19 is projected to hit particularly hard the food, nutrition, and health security of vulnerable groups including young children, pregnant and lactating women further exacerbating social and health inequities (Roberton et al., 2020; UNICEF, WHO, WFP, &FAO, 2020). One mechanism by which this is likely to happen is through a major decline in food security which exists when ‘all people, at all times, have physical, social, and economic access to sufficient, safe, and nutritious food which meets their dietary needs and food preferences for an active and healthy life’ (FAO, 2006). Indeed, ‘The [COVID‐19] pandemic may well devastate livelihoods and food security, especially in fragile contexts and particularly for the most vulnerable people working in the informal agricultural and nonagricultural sectors. A global recession will majorly disrupt food supply chains’ (Global Network Against Food Crises, 2020). Global evidence shows that this direct increase in food insecurity would be a serious public health concern as household food insecurity (HFI) has been shown to negatively affect caregiver mental health and that this in turn has a negative impact on early child development outcomes (Pedroso, Buccini, Venancio, Pérez‐Escamilla, & Gubert, 2020; Pérez‐Escamilla & Vianna, 2012) as young children cannot receive the nurturing care that they need (Nurturing Care for Early Childhood Development, 2020). HFI in early life has indeed been consistently associated with child internalization and externalization of problems, behavioural problems in school, and poor academic performance and intellectual outcomes once those children become school age (de Oliveira et al., 2020). HFI has also been associated with family chaos (Fiese, Gundersen, Koester, & Jones, 2016; Rosemond et al., 2019) and intimate partner violence (Diamond‐Smith, Conroy, Tsai, Nekkanti, & Weiser, 2019) and may be associated with suboptimal infant feeding practices, possibly related to perceived insufficient milk of food insecure women (Orr, Dachner, Frank, & Tarasuk, 2018; Webb‐Girard et al., 2012). HFI increases the risk of chronic undernutrition and infectious diseases in children, maternal anaemia, obesity (especially among adult women), and the development of noncommunicable diseases, including type 2 diabetes (FAO, 2019), which in turn are risk factors themselves for poorer prognosis in COVID‐19patients (Watanabe et al., 2020). This follows a clear syndemic paradigm (Singer & Clair, 2003), where two or more coexistent diseasesact synergistically to cause excess burden of disease in a population (Swinburn et al., 2019) and highlights how important it is that mitigation of HFI during the COVID‐19 pandemic be a national and global priority (Pérez‐Escamilla, 2017).

COVID‐19 is making access to and availability of food more challenging for many worldwide, an undesirable situation that is likely to remain for the foreseeable future as an aftershock of the pandemic (Poppik, 2020).Massive poverty increases, reductions in cross‐border trade and internal and external labour migration and employment have resulted in major food systems disruptions (International Panel of Experts on Sustainable Food Systems., 2020; Pollan, 2020). For example, in the United States, massive unemployment and loss of income has led thousands of families where young children live to become food insecure (Bauer, 2020) and need to queue at emergency food distribution centres. Furthermore, the infection of meat‐packing plant workers in the Midwest has led to the shutdown of such plants and subsequently to pork and poultry shortages nationwide (Pollan, 2020; The Economist, 2020a). In addition, the inability to transport produce from the fields to points of distribution has forced farmers to leave behind millions of tons of fresh unharvested produce rotten in the fields (Lush, 2020), and to dump large amounts of milk (Pollan, 2020). Many countries rely on farm workers, often migrant labourers, to plant and harvest crops (Purdy, 2020). COVID‐19 has strongly disrupted the movement of farm workers across countries also leading to a shortage of availability and access to staple foods and fresh produce (Purdy, 2020), which in turn may lead to an increased consumption of ultraprocessed foods and beverages. In lower‐income countries, a large proportion of families depend on income generated through the informal economy (International Labour Organization, 2020b). As a result of COVID‐19‐related movement restrictions, many of them have lost their income sources without having access to any social protection to properly support them during the crisis (The Economist, 2020b; Vilar‐Compte et al, 2020). As recently reported from South Africa and other lower‐income countries, populations are now more fearful of dying of hunger than of COVID‐19 itself (The Guardian, 2020) which is understandable given the tens of millions of families already been pushed into extreme poverty, severely limiting their capacity to purchasing basic foods, and the fact that food systems ‐ including supply chains ‐ in these settings are very unstable and weak compared with higher‐income countries, hence more likely to be decimated by the COVID‐19 pandemic (Global Nutrition Report, 2020 ). In addition, global food trade has been heavily affected as, in countries like India, ships have had to stay in ports without the ability to move basic staples such as rice (Gulf News, 2020). With COVID‐19‐related school closures occurring in nearly 200 countries across the world, more than 368 million school children are currently missing out on school meals according to the latest data from the World Food Programme (Van Lancker & Parolin, 2020; WFP, 2020). Approximately half of these children are in low and lower‐middle‐income countries. Lost access to school meals is not only impacting on HFI and threatening children's health, but it also affects the most vulnerable families by reducing their income, particularly in rural communities where small‐scale farmers represent an important ring in the schools' supply chain. By the same token, it is important to acknowledge that adolescents are also a highly vulnerable population during this pandemic as many of them rely on schools for their food and nutrition security and for some health services such as deworming.

A major concern is the increasing evidence of the high risk of major disruptions in maternal, newborn, and child health services (MNCH), particularly in low‐ and middle‐ income countries during COVID‐19 times (Roberton et al., 2020). This may be due in part due to fears related to seeking health care, but may also be due to limited transportation availability during lockdowns as well as limited health service provision as health facilities turn their attention to the pandemic. MNCH services have been responsible for strong reductions in maternal and infant mortality, as well as reductions in under‐nutrition, in the last several decades. These gains must not be lost and a strong focus on these and other important Sustainable Development Goals must continue in spite of the current global pandemic. Likewise, global interest in adolescent‐focused food and nutrition research and programmatic efforts has been growing substantially in recent times (Salam et al., 2019). Losing sight of this during the COVID‐19 pandemic would also take us backwards in this priority area.

There is no doubt that COVID‐19 has become a natural experiment illustrating how unprepared the world is to protect populations against hunger, food, nutrition, and health insecurity during global emergency situations. As a result of rapid unplanned urbanization, and climate change, these types of pandemics will likely be with us for the years to come (Jowell & Barry, 2020). Since 2009, the world has been affected by numerous pandemics including H1N1, SARS, MARS, Ebola, and currently COVID‐19. We now have an opportunity to rethink the dysfunctional global food system upon which the vast majority of the world now depends and reconfigure the types of programmes,policies, and multilevel intersectoral coordination mechanisms that are needed to ensure food and nutrition security for all, including young children,adolescents,pregnant and lactating women (UNICEF, WHO, WFP, & FAO, 2020). We urge donors to fund research that focuses on food and nutrition implications of pandemics and answers emerging implementation science questions around effective delivery of equitable social protection programmes and policies in these "unusual" circumstances. Research recommendations for moving forward include how to: (1) adapt and continue school feeding and other food assistance programmes to continue the provision of meals to quarantined families with children, adolescents,pregnant and lactating women; (2) develop equitable effective rapid response systems to prevent or mitigate food insecurity based on complex adaptive systems frameworks (Barnhill et al., 2018; Paina & Peters, 2012) that address the food, nutrition, health care, and social protection systems, and the multiple complex interrelationship among them; paying special attention to households with children, adolescents, pregnant and lactating women; and (3) monitor and use surveillance systems to effectively identify and target provision of healthy and nutritious foods to families that are the most socio‐economically vulnerable, such as young children,adolescents, pregnant and lactating women. It is our hope that addressing these questions will protect the food and nutrition security as well as the health, and wellbeing of children and youth,pregnant and lactating women, and their families (Nurturing Care for Early Childhood Development, 2020) in the complex syndemics world in which we now live. The ripple effects of the COVID‐19 pandemic will be negatively affecting the food and nutrition security, health, and well‐being of families with young children and pregnant and lactating women for years to come (GAIN, 2020; World Vision, 2020), hence decisive action informed by sound implementation science research is needed now! Not doing so will not only put the development of individuals at further risk, but also negatively impact on the development potential of entire nations and ultimately the sustainability of our planet (Jowell & Barry, 2020; Pérez‐Escamilla, 2017; Swinburn et al., 2019).

## CONTRIBUTIONS

All authors have read and approved the final manuscript. RP‐E wrote the first draft, and KC and VHM reviewed and provided feedback that led to substantive changes to original draft.
